# Mechanics

**Published:** 1874-04

**Authors:** 


### MECHANICS.

A MAN who works hard for his living is no better on that account than the man who can do without work. The occupation of a man does not give him character; it is the character of the workman which dignifies the calling. At the same time, we see a man working day after day, year in and year out, to make a comfortable living for his family, whether by handling the saw, shoving the plane, or wielding the sledge-hammer, and coming home at night weary, and soiled, and begrimed; it is to his credit, and it does give him character when it is taken into account that he thus bravely works for an honest living, rather than abandon himself to idleness and crime—making him really one of Nature's noblemen.

